# Estimating the Biases Associated with Self-Perceived, Self-Reported, and Measured BMI on Mental Health

**DOI:** 10.1371/journal.pone.0081021

**Published:** 2013-12-04

**Authors:** Mir M. Ali, Travis Minor, Aliaksandr Amialchuk

**Affiliations:** 1 Substance Abuse and Mental Health Services Administration, Analysis and Services Research Branch, Rockville, Maryland, United States of America; 2 Food and Drug Administration, Office of Regulations, Policy and Social Science, College Park, Maryland, United States of America; 3 University of Toledo, Department of Economics, Toledo, Ohio, United States of America; University of Sao Paulo, Brazil

## Abstract

**Purpose:**

The purpose of the study is to explore the relationship between individuals' perceptions of their weight-status, self-reported height and weight, and measured weight status.

**Methods:**

A national survey of 9,248 adolescents (47% male) between the ages of 11 and 27 is analyzed to determine whether inaccuracies in reporting are caused by misperception or conscious intent, and whether there tends to be a systematic bias in how individuals self-report. Self-esteem was used as an example of an important outcome variable in order to illustrate the magnitudes of the biases that may arise when using different measures of body size.

**Results:**

Our results indicate that measured obesity status is associated with the reduction in Rosenberg Self-Esteem (RSE) of 0.30 points (p-value 0.005) among adolescents and 0.20 points (p-value 0.002) among young adults; in addition, using self-reported height and weight as opposed to measured height and weight does not result in a statistically detectable difference in the estimates.

**Conclusions:**

Individuals' self-reports of height and weight are not as unreliable as we might have expected. Although estimates from measured height and weight are preferred, in the absence of such measures, self-reported measures would likely be a reliable alternative. The differences in self-perception of weight status, however, imply that it is not comparable to measured weight categories.

## Introduction

Excess body weight among children and adults has been documented widely over the last two decades and is considered one of the most pressing public health concerns today [1]; [2]. Although the literature on obesity is vast, a clear consensus on its measurement does not exist [3]. Weight status classification based on Body Mass Index (BMI) is used extensively but the literature varies with regard to the use of measured versus self-reported BMI [4]. An emerging literature has also explored the importance of self-perception of weight, especially among adolescents [5]; [6] and found that self-perception is not only more important than actual weight in determining some health outcomes [7], it is also quite prevalent and in many cases correlated with many weight related behaviors [8]. Thus, there is a current need for the study of the relationship among these different measures of body weight, and the magnitudes of biases associated with the use of these different measures in empirical applications.

A number of studies have explored the relationship between self-perception and objective measures of weight [3], [9]. However, none of these studies have examined the differences in self-perceived weight classification, weight status based on self-reported height and weight, and weight status based on measured height and weight. The purpose of this study is to explore the relationship between individuals' perceptions of their weight status, weight status based on self-reported height and weight, and weight status based on measured height and weight. Information on body weight from these different sources allows us to determine whether there tends to be a systematic bias in how individuals self-report.

To document the extent of such differences we utilize a nationally representative data set that contains the individuals' measured height and weight, self-reported height and weight, and self-perception of body weight during the individuals' adolescence and young adulthood. We examine, how these various measures of body weight impact an individuals' self-esteem, which has been consistently documented to be correlated with actual body weight and self-perception of body weight [10]; [11].

## Methodology

### Ethics Statement

We are registered and approved users of the Add Health dataset. As a part of the process for acquiring the data we underwent IRB review and received approval from the Institutional Review Board of the University of Toledo (2007). We are in no way using human or animal subjects directly (we are analyzing pre-existing data), thus written consent was not necessary. We have successfully completed our training on human subjects research review as well as HIPAA.

### Data

We utilize panel data from the National Longitudinal Study of Adolescent Health (Add Health). Add Health consists of data on adolescents in 132 schools nationwide between grades 7 to 12. The in-school portion of the first wave of the survey (1994) contains a cross-section of data on about 90,000 adolescents. A subset of the initial sample (20,745 respondents) was also interviewed in their homes (in-home portion of the data) with follow-up surveys in 1996 and in 2002, when most respondents had made a transition to adulthood. The primary data for our analysis come from the last two waves (1996 and 2002) of the in-home survey portion of Add Health. A key feature of the data set is that it contains information on not only the respondent's height and weight as measured by the interviewers in both the waves, but it also contains their self-reported height and weight as well as their self-perception of weight status for the waves. In addition, one parent (mostly mothers) for each adolescent was interviewed as part of the in-home parent survey in 1994. This parent survey is our primary source of controls for family characteristics. The analysis sample includes all individuals who were interviewed in both waves of the survey with non-missing information on body weight measures and other analysis variables (N = 9,248). It is important to note that replacing the missing values with the mean and including it in the analysis did not change our results substantially.

### Outcome Variables

Our outcome measure is the abridged Rosenberg Self-Esteem (RSE) Scale [12]. Add Health administered six of the ten questions typically used to measure the full RSE scale. For example, respondents were asked whether they felt “socially accepted”, whether they had “a lot to be proud of” and whether they liked “themselves the way they are”. Responses by the adolescents were: strongly agree ( = 5); agree ( = 4); neither agree nor disagree ( = 3); disagree ( = 2); or strongly disagree ( = 1). These responses are summed to produce a score of 6 to 30, with a higher score indicating greater self-esteem [5]; [13]; [14].

### Explanatory Variables

The main exploratory variables of interest is the individuals weight status based on three sources – interviewer-measured height and weight (*measured*); self-reported height and weight (*self-reported*); and self-perception of weight status (*self-perceived*). Our actual weight measure was calculated using the Centers for Disease Control and Prevention (CDC) growth charts for 2000, which are based on the adolescent's BMI [*weight(kg)/height(m^2^)*] relative to the national distribution and are age- and gender- specific. Adolescents (i.e. the respondents in wave II, average age 16) who are at the 95^th^ percentile or above are classified as being obese. This is the CDC recommended criteria for defining obesity for ages 2–19, which covers every respondent in wave II of the survey. We utilize similar CDC cutoffs for weight status based on the adolescents' self-reported height and weight. To measure the weight status when these adolescents have transitioned into young adulthood (i.e. the respondents in wave III, average age 21) we adopt a different CDC recommended cutoff for all individuals aged 20 and over: individuals with BMI above 30 are classified as being obese. Add Health also asked the respondents to indicate what they thought of themselves in terms of weight. Our self-perception of obesity was constructed based on an individual indicating they thought of themselves as “very overweight”.

Other control variables include demographic characteristics such as age, grade, whether the individual is first born, whether the individual have any siblings and whether he/she was born in the United States. We also include a self-reported indicator of good health and whether the individual considered himself/herself to be religious. The parent survey of Add Health allows us to control for a number of parental characteristics including, parental education, household income, whether any of the parent is a welfare recipient and whether the parents choose the area of their residence because of the school district. Variables from the parent survey are also used to create measures of both the parents' obesity status, the individual's birth weight and whether the individual was breast fed.

### Statistical Analyses

The purpose of this paper is to compare the estimated effects of actual versus self-reported obesity status. We use RSE, a measure of mental health, as the outcome of interest and test three separate measures of obesity. The first measure is calculated from a respondent's *measured* height and weight; this should be the most reliable measure. The second measure of obesity is calculated from a respondent's *self-reported* height and weight, and the third measure is simply a respondent's *self-perception* of their weight category. The relationship is modeled as: 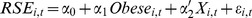
where *RSE_i,t_* is an ordinal variable that rises with person *i*'*s* self-esteem; *α_0_* is a constant; *Obese_i,t_* is a one of three indicator variables measuring person *i*'s weight status in year *t*; *X_i,t_* is a vector of person-year specific controls; and *ε_i,t_* is the idiosyncratic error term. Multivariate estimation is carried out using Ordinary Least Squares regression.

While the results for each of the three indicators for obesity are interesting, we are more concerned with how closely the *self-reported* and *self-perceived* measures approximate the *measured* effect of obesity. Therefore, we employ a Chow [15] test to examine whether the differences in estimates are statistically significant from one another. The null-hypothesis is no statistical difference between *measured* and either *self-reported* or *self-perceived* obesity.

## Results

Table 1 reports summary statistics for the outcome measure and all of the control variables used in our analysis. Table 2 presents a detailed comparison of the *measured* and *self-reported* or *self-perceived* weight categories. Looking at the top left quadrant, where *measured* and *self-reported* are compared in wave II, we observe 98.56 percent of non-obese respondents accurately identifying themselves. Similarly, 79.33 percent of obese respondents correctly identify themselves as obese. However, we also see that 1.44 percent of respondents incorrectly classify them as obese, when in fact they are not obese, and 20.67 percent of obese respondents incorrectly identify as non-obese. When we look at *measured* compared to *self-perceived* category in wave II, located in the lower left quadrant, we observe somewhat different results with respect to incorrectly identifying own body classification. Here, we see that only 1.01 percent of non-obese respondents are incorrectly identifying as obese; however, a much larger portion, 79.33 percent of obese respondents, now incorrectly classify themselves as non-obese. Moving to wave III, we observe a similar effect. More respondents incorrectly identify themselves in the *self-perceived* body category than they do with the *self-reported* height and weight measures. In wave III, 79.01 percent of obese respondents incorrectly perceive themselves as non-obese; while only 28.35 percent of those same obese respondents would be incorrectly classified based on their self-reported height and weight.

**Table 1:** Summary Statistics.

	Wave II	Wave III
**Key Variables**		
Mean Mental Health Score (range, 5–30)	25.1	21.0
Measured Obesity (%)	12.6	25.4
Self-Reported Height/Weight (%)	11.3	19.6
Self-Perceived Category (%)	3.5	5.9
**Demographic Controls**		
Male (%)	47.4	47.4
Hispanic (%)	15.1	15.1
Black (%)	20.1	20.1
White (%)	59.7	59.7
Born in US (%)	72.9	72.9
Sibling (%)	81.4	81.4
Mean Age (range, 11–27)	16.0	21.5
7th Grade (%)	0.9	--
8th Grade (%)	15.6	--
9th Grade (%)	16.4	--
10th Grade (%)	18.8	--
11th Grade (%)	19.9	--
12th/graduate (%)	17.9	87.6
Welfare (%)	24.4	24.4
Chose School District (%)	47	47
Mom College Edu. (%)	1.3	1.3
Dad College Edu. (%)	3.1	3.1
Mom Obese (%)	18.4	18.4
Dad Obese (%)	9.9	9.9
Good Health (%)	94	95.6
Religious (%)	59.4	85.2
Mean Birth Weight, lb (range, 3–12)	6.7	6.7
Breast Fed (%)	46.9	46.9
First Born (%)	49.7	49.7

*Notes*: n = 9,248 observations.

**Table 2:** Detailed Comparison of Measured and Self-Described Weight Categories.

		Measured Weight			
		Wave II		Wave III	
		Non-Obese	Obese	Non-Obese	Obese
**Self-Reported Ht./Wt.**	Non-Obese	98.56%	20.67%	98.13%	28.35%
		(7966)	(241)	(6770)	(666)
					
	Obese	1.44%	79.33%	1.87%	71.65%
		(116)	(925)	(129)	(1683)
**Self- Perceived Category**	Non-Obese	98.99%	79.33%	99.22%	79.01%
		(8000)	(925)	(6845)	(1856)
	Obese	1.01%	20.67%	0.78%	20.99%
		(82)	(241)	(54)	(493)
	**Total Obs.**	**8,082**	**1,166**	**6,899**	**2,349**

*Notes*: n = 9,248 observations. Percentages are calculated out of the total within each measured weight category. Total number of observations within each category is presented in parenthesis. “Ht.” – height, “Wt.” - weight”

Results of the statistical analysis, outlined above, should show the effect of actual, self-reported, or self-perceived obesity status on mental health of a respondent when he/she is as an adolescent and a young adult. We ran similar estimations with additional indicators for being underweight or overweight, as well as being obese; results are qualitatively similar and are available from the authors upon request. We focus on obesity in the main analysis, because it is of primary concern with regards to public health and for ease of interpretation of the results.

Table 3 presents the estimated effect of obesity status on mental health measure (RSE), in waves II and III. *Measured* obesity is estimated to reduce a respondent's RSE score by 0.309 points in wave II, where respondents are younger and still in high school, and by 0.204 points in wave III. *Self-reported* obesity shows similar negative effects of 0.257 points in wave II and 0.269 points in wave III. Finally, *self-perceived* indicates a higher penalty for classifying oneself as obese. In wave II, *self-perceived* obesity is estimated to reduce a respondent's RSE score by 1.786 points. The effect is smaller in wave III, where *self-perceived* obesity causes a reduction of 1.103 points.

**Table 3:** The Estimated Effect of Obesity on Mental Health.

	Wave II			Wave III		
	Measured Weight	Reported Ht./Wt.	Perceived Category	Measured Weight	Reported Ht./Wt.	Perceived Category
Obese	−0.309***	−0.257**	−1.786***	−0.204***	−0.269***	−1.103***
	(0.109)	(0.115)	(0.194)	(0.0662)	(0.0727)	(0.121)
R^2^	0.073	0.073	0.081	0.049	0.049	0.056
F-stat	29.23	29.10	32.54	23.73	23.95	27.61
p-val.	(0.000)	(0.000)	(0.000)	(0.000)	(0.000)	(0.000)

*Notes*: n = 9,248 observations. ** and *** represent significance at the 5, and 1 percent levels, respectively. Numbers in parenthesis are standard deviations, except where otherwise noted. “Ht.” – height, “Wt.” - weight”

Table 4 presents the results of a Chow test of the difference between the *self-reported* or *self-perceived* and *measured* obesity coefficients (with adequate power of the test of at least 90%). In wave II, we observe no statistical difference in coefficients between *self-reported* and *measured* obesity. This finding is not repeated when we compare the coefficients of *self-perceived* and *measured* obesity. Test results indicate a rejection of the null-hypothesis; meaning *self-perceived* obesity is not a valid proxy for *measured* obesity. Wave III shows quantitatively similar results.

**Table 4:** Chow Test of the Effect of Actual Vs. Reported Obesity.

	Wave II		Wave III	
	Reported Ht./Wt.	Perceived Category	Reported Ht./Wt.	Perceived Category
Obese	0.55	49.28***	1.52	50.44***
p-val.	(0.460)	(0.000)	(0.217)	(0.000)

*Notes*: n = 9,248. *** represents significance at the 1 percent level. The null hypothesis of the Chow Test is no difference between actual and self-reported weight categories. “Ht.” – height, “Wt.” - weight”

## Discussion

Statistics from Table 2 suggest that individuals may be more accurate with self-reporting their height and weight than with the self-perception of body weight classification. These statistics suggests some noise in both of these measures of obesity; however, they also suggest that *self-perceived* weight category has a more systematic bias than *self-reported* height and weight alone. Further evidence of this is found upon examination of the regression coefficients from Table 3. While the differences in the estimates on *measured*, *self-reported*, and *self-perceived* obesity are interesting, we need to more formally test whether there is an actual difference in the estimated coefficients before we can draw any conclusions about their usefulness.

Results from Table 4 indicate that *self-reported* and *measured* obesity are explaining the same variation in the outcome, in wave II. Additionally, in wave III, *self-reported* obesity is not statistically different from *measured* obesity, indicating that the two could be used somewhat interchangeably; however, *self-perceived* obesity is estimated to be significantly different from *measured* obesity. This suggests that, although *self-reported* may be an appropriate alternative to *measured* obesity status, *self-perceived* does not capture that same variation in the mental health outcome examined, for either adolescents or young adults. It is also interesting to note that the magnitude of each test statistic, a potential indicator of the degree of variation between these and the *measured* effect, is larger in wave III. This may indicate that, as respondents age, they are more likely to misrepresent their weight status, both in terms of the reported height and weight and the perceived weight status.

The differences in self-perception of weight and clinical weight categories imply that even though individuals are accurate in reporting their height and weight, they are not accurate with respect to perceived weight categories. More interestingly, we see that such differences between self-perception and clinical weight classification become larger as the individuals transition into adulthood. One might expect that, as individuals transition into adulthood, self-perception and clinical weight status would begin to converge; however, our results indicate the opposite and highlight the importance of self-perception of weight when studying psychological outcomes. Results also indicate that policy measures enacted to create awareness about a healthy body image are important for adolescents and adults alike.

## Conclusion

Using a nationally representative sample of adolescents, we investigated the differences in the relationship between body weight and self-esteem using three different measures of body weight. Consistent with the previous literature [16], [17], we find that higher body weight is negatively correlated with self-esteem, and estimates vary between measured and self-reported or self-perceived obesity. Specifically, differences in self-perceived weight status and clinical categories of weight status are more pronounced when compared to weight status based on measured and self-reported height and weight. Additionally, these differences become larger as individual transitions into young adulthood.

Our results indicate that using self-reported height and weight or measured height and weight does not result in a statistically detectable difference in the estimates for mental health. This could mean that individual's self-reported height and weight are not as unreliable as we might have expected. Although estimates from measured height and weight are preferred, in the absence of such measures, self-reported measures are a reliable alternative.

As with any empirical strategy, our approach is subject to criticism and it is prudent to regard our results as demonstrating a strong association rather than a causal relationship. Also, it is possible that individual's self-esteem could influence their own body perception rather than the other way around. However, due to the fact that reverse causality is present in most outcomes correlated with obesity, our choice of self-esteem is primarily driven by the extensive literature that documents a causal relationship between body weight and self-esteem. Future research should explore whether the differences in estimates are also present in other outcomes.
